# Quantification of the Number of Steps in a School Recess by Means of Smart Bands: Proposal of Referential Values for Children and Adolescents

**DOI:** 10.3390/children10060915

**Published:** 2023-05-23

**Authors:** Jose Sulla-Torres, Rubén Vidal-Espinoza, Christopher Avendaño Llanque, Alexander Calla Gamboa, Manuel Zúñiga Carnero, Marco Cossio-Bolaños, Rossana Gomez-Campos

**Affiliations:** 1 Escuela de Ingeniería de Sistemas, Universidad Católica de Santa María, Urb. San José, San Jose s/n, Yanahuara, Arequipa 04001, Peru; 2 Escuela de Educación Inicial, Departamento de Educación Diferencial, Facultad de Educación, Universidad Católica Silva Henriquez, Santiago 8330225, Chile; 3 Carrera de Ciencias de la Actividad Física y del Deporte, Facultad de Ciencias de la Salud, Universidad San Ignacio de Loyola, Av. La Fontana 550, La Molina, Lima 15024, Peru

**Keywords:** steps, school recess, reference values, smart band

## Abstract

(1) Background: Regular physical activity has multiple benefits. Therefore, school recess is a key tool to provide opportunities for schoolchildren to engage in extracurricular physical activity, have fun, play and interact with their peers. The aim is to provide reference data to quantify the number of steps that children and adolescents perform in a school recess using smart bands according to age range and sex. (2) Method: A descriptive cross-sectional study was carried out in 494 schoolchildren aged 6 to 17 years (292 males and 202 females). Weight, standing height and waist circumference (WC) were evaluated. The body mass index (BMI) was calculated. The quantification of the number of steps during school recess was performed using a smart band. (3) Results: Percentiles were constructed for the number of steps (number of steps/recess). The cut-off points considered were <p25 (below average), p25 to p75 (average) and >p75 (above average). The median values in both sexes decreased as the age range increased. Youth who walked fewer steps during recess (<p25: below average) had elevated BMI and WC values relative to those who walked within average and above average. (4) Conclusion: The number of steps taken by schoolchildren during school recess decreases drastically with advancing age. The proposed reference values can be used to categorize schoolchildren according to the number of steps taken and to compare them among their peers. The results suggest their use and application in schools as a way of achieving the minimum physical activity recommendations.

## 1. Introduction

Regular physical activity (RPA) promotes growth and development in children and adolescents and has multiple physical, mental and psychosocial health benefits [1,2]. It also positively affects body composition, i.e., higher levels of physical activity are associated with a healthy weight status in children and adolescents [3].

RPA is one of the most effective ways to prevent cardiovascular and mental diseases, as well as to improve physical fitness in children and adolescents [4]. Its assessment in the school stage is relevant, since it helps in the regulation and quantification of daily physical activity levels [5].

In fact, among the most commonly used methods are subjective measures (self-report, such as questionnaires, and proximity reports from parents and teachers), as well as objective measures (such as heart rate, accelerometry, pedometers, direct observation and doubly labeled water) [6].

Recently, smart bands have become part of people’s daily lives, since they can be used as an objective method that allows to evaluate factors directly on the body [7], where they continuously monitor and record data in real time, such as physical activity, energy consumption and sleep quality [8], among other parameters.

In recent years, several studies have prioritized the measurement and monitoring of physical activity patterns by counting steps per day in free-living conditions in children and adolescents in various regions of the world using pedometers [9,10,11,12] and accelerometers [13,14,15,16,17]. However, studies that have quantified the number of steps during school recess are scarce [18,19,20]. It is widely known that recess is a part of the school day that allows children and teachers to rest from classes [1].

Recess is also characterized as a potentially important time, and during which children can engage in daily physical activity. For many children, it represents the main opportunity to engage in physical activity during the school day [19], as well as to interact outdoors among peers [21].

In general, regardless of the performance of physical activity during recess, scientific evidence suggests that the practice of RPA is associated with high levels of physical fitness (e.g., cardiovascular endurance, muscular strength and endurance, flexibility and body composition), which lead to the preservation of better physical, mental and social health status among children and adolescents [22,23,24].

In this context, the Center for Disease Control and Prevention (CDC) and the American Society of Health and Physical Educators SHAPE [25] recommend at least 20 min as a rest period within the school day, although the scheduling and duration of school breaks may vary between countries and geographic regions [26]. Therefore, an analysis of children and adolescents’ leisure time activity should be carried out, taking into account the cultural, socioeconomic and climatic environment [18], respectively.

Therefore, based on the fact that there is freedom in establishing a prudent time for a school recess, the Ministry of Education of Peru in its guidelines for the development of schedules in educational institutions suggests about 20 min [27], although some schools generally set their recesses at 15 min. This indicator could help to identify the number of steps that children and adolescents take.

This information could serve as an effective motivational tool and a way to monitor and promote the accumulation of RPA in schools and consequently allow the achievement of the minimum physical activity recommendations [28].

In fact, the pedometer is an inexpensive, unobtrusive and ideal measurement tool for large populations. It can be used primarily to monitor and quantify the short and frequent periods that occur during the school day. It can even be used to plan and evaluate school-based physical activity intervention programs.

Therefore, the main objective of this study was to provide baseline data to quantify the number of steps taken by children and adolescents in a school recess using smart bands according to age range and sex.

## 2. Materials and Methods

### 2.1. Type of Study and Sample

A descriptive cross-sectional study was carried out in 494 schoolchildren (292 males and 202 females) with an age range of 6 to 17 years (13.7 ± 2.9 years). The sample selection was non-probabilistic (convenience). Two state schools in the city of Arequipa (Peru) were evaluated. This city is located south of Lima (capital of Peru) at 2320 m above sea level. The schoolchildren attended physical education classes twice a week, each lasting 45 min.

In order to carry out the study in both schools, permission was requested from the administration of each school. Then, parents and/or guardians were informed about the objective of the project. Parents who agreed to participate in the study signed the informed consent form to authorize their children’s participation. On the day of the evaluation, the children and adolescents signed the informed assent.

Schoolchildren from 6 to 17 years of age were included. Schoolchildren who were on medical rest and those who could not perform any type of physical activity during school hours due to medical prescription were excluded from the study.

Anthropometric measurements and the use of the smart band were applied according to the suggestions described by the local ethics committee (UCSM-096-2022) and the Declaration of Helsinki (World Medical Association) for human beings.

### 2.2. Techniques and Procedures

Anthropometric measurements were evaluated at each school’s facilities. The evaluation team consisted of 4 experienced physical education teachers. Weight and standing height were evaluated using the standardized protocol of Ross and Marfell-Jones [29]. Body weight (kg) was evaluated using an electronic scale BC-730 (Tanita Corporation, Tokyo, Japan), United Kingdom with a scale from 0 to 150 kg and an accuracy of 100 g. Standing height was measured according to the Frankfurt plane using a portable stadiometer (Seca 216, Gmbh and Co. KG, Hamburg, Germany) accurate within 0.1 mm. Waist circumference (WC) was measured using a tape measure (Seca) to the nearest 1 mm. The body mass index (BMI) was calculated using the following formula: BMI = weight (kg)/height^2^ (m). According to the BMI Z-score, patients were classified as follows: underweight/normal weight with Z-scores between −2 and +0.99, overweight from 1 to 1.99, obese from 2 to 2.99 and very obese ≥3 [30].

To categorize abdominal adiposity (WC) by age and sex, the suggestions described by Fernández et al. [31] were used. It was categorized into two groups (without risk < p75 and with risk > p75).

The quantification of the number of steps during school recess was performed using a smart band (Huawei band 7, China) with an AMOLED screen of 194 × 368, and with 1.47 inches. This band marking had been previously used in other studies [32]. Each child and adolescent were fitted with the smart band on the wrist of the right hand before recess. They were instructed to go about their daily activities as normal during the 15 min of recess. After recess was over, the evaluators approached each child to remove the smart band. A daily one-week school break was evaluated.

### 2.3. Statistical Analysis

All data were normal and verified using the Kolmogorov–Smirnov (K-S) test. Descriptive statistics (mean and standard deviation) were calculated. Differences between both sexes were calculated by means of the *t*-test for independent samples. The LMS method [33] was used to construct the percentiles. The curves L, M and S represent skewness (lambda), median (mu) and coefficient of variation (sigma). The LMS method uses the Box–Cox transformation to fit the data distribution to a normal distribution by minimizing the effects of skewness. The L, M and S parameters were calculated according to the maximum penalized method [34]. Data processing was performed using LMS Chartmaker Pro software (The Institute of Child Health, London, UK) [35]. Differences between BMI and WC values according to step/recreation categories were determined via one-way ANOVA and Tukey’s test of specificity. In all cases, *p* < 0.05 was considered to be significant. Calculations were performed in Excel spreadsheets and SPSS 18.0.

## 3. Results

The variables characterizing the children and adolescents studied are shown in Table 1. In terms of body weight, there were no differences between both sexes from 6–7 years old to 12–13 years old (*p* > 0.05); then, at 14–15 years old and 16–17 years old, males presented a higher body weight (*p* < 0.05). In terms of height, there were no differences from 6–7 years to 10–11 years (*p* > 0.05). However, from 12–13 years to 16–17 years, males presented a taller height than females (*p* < 0.05). In terms of BMI, there were no significant differences in all age ranges between both sexes (*p* > 0.05).

In terms of WC, males presented a higher WC than females at 8–9 years and at advanced ages (12–13 years, 14–15 years and 16–17 years), while there were no differences at 6–7 years and 10–11 years (*p* > 0.05).

Regarding the number of steps taken in the 15 min recess, males took more steps than females from 6–7 years old to 12–13 years old (*p* < 0.05). Then, at older ages (14–15 years and 16–17 years), no differences were observed between both sexes (*p* > 0.05). There were no significant differences in mean and maximum HR between both sexes (*p* > 0.05).

**Figure 1:**
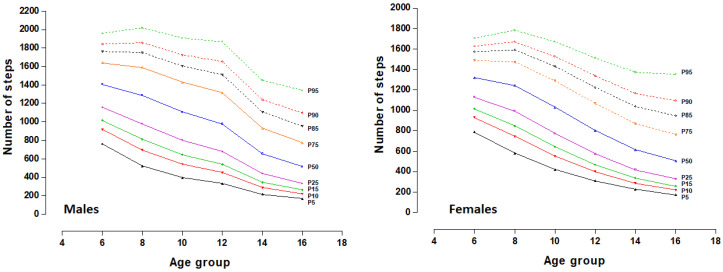
Distribution of percentiles for the number of steps in a school recess in both sexes.

The distributions of the percentiles (P3, P5, P10, P25, P50, P75, P90, P95 and P97) for the number of steps/recreation are shown in Table 2 and Figure 1. The median values in both sexes decrease as the age range increases. For example, the number of steps at 8–9 years decreases by 8.4% in males and 5.9% in females; at 10–11 years, it drops for 21.2% in males and 22.0% in females; at 12–13 years, it decreases by 30.5% in males and 39.2% in females; at 14–15 years, it decreases by 53.3% in males and 53.5% in females and at 16–17 years, it decreases by 63.4% in males and 61.8% in females.

The BMI and WC values according to the categories of the number of steps during school recess are shown in Table 3. In the comparison of BMI in both sexes, there were no differences between youth categorized as below-average steps versus above-average steps (*p* > 0.05) and between the category average steps versus above-average steps (*p* > 0.05). However, there were differences in BMI between the categories of below-average steps versus above-average steps (*p* < 0.05).

In relation to WC, men categorized as below-average steps presented higher WC values in relation to their peers categorized as average and above-average steps (*p* < 0.05). In females, differences were observed among the three categories (*p* < 0.05), where WC increased as the number of steps decreased.

**Figure 2:**
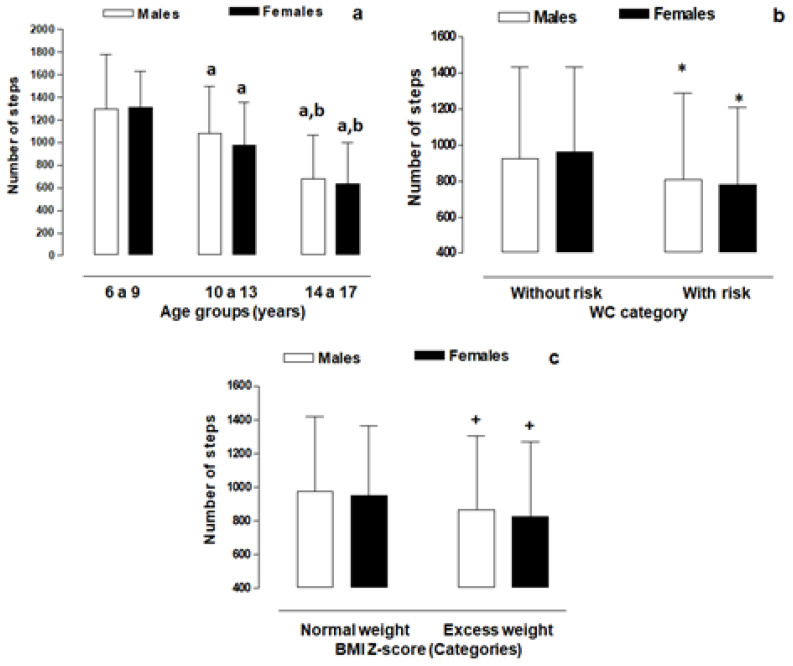
Comparison of the number of steps by age ranges, WC categories and BMI categories in both sexes. Legend: a: significant difference in relation to the group of 6 to 9 year olds; b: significant difference in relation to the group of 10 to 13 year olds; *: significant difference in relation to the category of without risk; +: significant difference in relation to the category of normal weight.

Figure 2a shows the comparison of the number of steps in three age groups. There were no significant differences between both sexes in each of the age categories (*p* > 0.05). However, we observed significant differences between the three categories and in both sexes (*p* < 0.05). This is evidence that the number of steps gradually decreases in each age category. At older ages (14 to 17 years), the number of steps decreases drastically to 677 ± 388.9 steps in males and 638.2 ± 360.9 steps in females.

Figure 2b shows the comparisons of the number of steps according to WC categories in both sexes. When we compared the number of steps between WC categories with and without risk, we found significant differences in both sexes (*p* < 0.05). For example, males (923.6 ± 510.4 steps) and females (963.0 ± 470.8 steps) categorized as non-risk performed a higher number of steps than their non-risk counterparts (males 884.8 ± 478.3 steps and females 782.9 ± 426.5 steps). Furthermore, when we compared between males and females in the same category (with risk and without risk), there were no significant differences (*p* > 0.05). The prevalence of abdominal adiposity with risk (according to the WC category) was 23.9% in both sexes (in males 15.4%, *n* = 76, and in females 8.5%, *n* = 42).

Figure 2c shows the comparisons of the number of steps in both sexes according to BMI categories (Z-Score). There were no significant differences between males and females in the normal weight category (males: 975.2 ± 444.5 steps, and females: 950.3 ± 411.2 steps) and in the overweight category (males: 865.1 ± 440.0 steps, and females: 823.4 ± 442.2 steps). However, we observed significant differences (*p* < 0.05) in the number of steps between males categorized as normal weight (975.2 ± 444.5 steps) versus overweight (865.1 ± 440.0 steps) and in females categorized as normal weight (950.3 ± 411.2 steps) versus overweight (823.4 ± 442.2 steps). The prevalence of nutritional status was 57.7% normal weight (males (33.4%, *n* = 165), females (24.3%, *n* = 120)) and 42.3% overweight (males (25.7%, *n* = 127), females (16.6%, *n* = 82)).

## 4. Discussion

The objective of the study was to provide reference data to quantify the number of steps that children and adolescents performed in a school recess using smart bands according to age range and sex.

The results of the study have evidenced that the number of steps children perform in a 15 min school recess range on average from 1406.4 to 504 steps in both sexes. Furthermore, we verified that the number of steps in both sexes decreases rapidly with increasing age, being more pronounced in females than in males. For example, during adolescence (at 14–15 years of age), they decreased by ~53.3% in males and ~53.5% in females, which evidently is a clear sign that physical activity patterns start to decrease during adolescence, drastically decreasing the physical activity volumes [36].

This information could help in the promotion of physical activity during school recess periods to develop strategies to encourage adequate physical activity levels [37,38].

In fact, the percentiles proposed in this study are a valuable tool that will allow for a cross-sectional assessment of the number of steps that children and adolescents perform during a 15 min school recess. This information can be useful for physical education teachers, parents and health professionals in general. It is widely known that the reference values proposed here can help to follow the trajectories of physical activity during school breaks at primary and secondary school level.

In this context, this study adopted the cut-off points of some recent research [39,40], where authors have proposed percentiles of the number of steps in a day. These can be interpreted as <p25 as below average, p25 to p75 as average and >p75 as above average.

In essence, percentiles based on step counts, regardless of the method used, can be applied to set targets for improvement, especially with those children and adolescents below the 25th percentile [39]. For example, children and adolescents in this study categorized below p25 (number of steps/recreation) evidenced higher values of BMI and WC relative to their counterparts who performed a higher volume of steps during a 15 min recess. Additionally, we verified that children and adolescents categorized according to WC with risk showed lower values in the number of steps compared to their counterparts without risk who reflected a higher number of steps.

In essence, the relationships made between BMI and WC with the number of steps have shown negative associations, confirming the importance of maintaining adequate values of BMI and WC.

This is clear evidence that the practice of free RPA in a 15 min school recess can have important repercussions on the weight status of the schoolchildren studied. In fact, some recent studies support the notion that the practice of physical exercise, both of a short duration as well as on a regular basis, allows children and adolescents to reduce their amount of fat, reduce their body weight and improve their cardiovascular function [41,42].

In addition, recently, the World Health Organization (WHO) [43] has recommended that all countries establish national guidelines and implement appropriate national and subnational physical activity policies and programs to enable people of all ages and abilities to be physically active and improve their health.

In general, the reference values proposed here can serve to motivate people to be physically active [40]. Childhood and adolescence are the best stages of human development to form adequate eating habits and maintain a healthy lifestyle, including the practice of physical activity [42].

In this sense, schools in recent years have become the focus of physical activity interventions among researchers, especially when aiming to quantify the time that children spend in this environment [44,45].

Therefore, it is important to individually monitor and diagnose physical activity patterns in children and adolescents [46]. Thus, school recess periods not only of a day but also of a week could provide accurate information on physical activity volumes expressed by one’s number of steps.

Thus, children and adolescents can accumulate up to 40% of their daily time doing physical activity during recess [47]. Therefore, it is counterproductive to suspend recess or replace it with classroom activities as punishment [2]. This limits the opportunity to participate in daily physical activity, preserve healthy bodies and enjoy movement, regardless of the times and seasons of the year, infrastructure and climate.

This study presents some potentialities and weaknesses that deserve to be analyzed. For example, the use of smart wristbands plays an important role in the field of health care and can be better integrated into people’s daily lives [7]. Their corresponding inclusion in the school setting can be considered, as they provide convenience and safety in monitoring physiological data generated by body movement in relation to other equipment and technologies. Additionally, as far as is known, this is the first study that has been carried out in Peru and in South America, whose information can serve as a baseline for future comparisons based on longitudinal studies, as well as for comparison with other realities and sociocultural contexts.

We also emphasize that this study has some weaknesses, which have to do with the type of sample selection. Therefore, future studies should calculate the sample size in a probabilistic way to achieve possible generalizations of the results. In addition, the cross-sectional design used in this study does not allow for causal relationships to be formulated; so, future studies should plan to carry out longitudinal studies. It is also suggested that it is necessary to monitor not only one school recess, but also all of the school recesses within a week.

## 5. Conclusions

This study concludes that the number of steps taken by children and adolescents during a 15 min school break decreases drastically with advancing age. Schoolchildren decreased their volume of steps by more than 50% around the age of 14–15 years. The reference values proposed in this study can be used to categorize schoolchildren according to the number of steps taken and to compare them among their peers. The results suggest their use and application in schools as a way of achieving the minimum recommendations for physical activity during school recess.

## Figures and Tables

**Table 1:** Anthropometric, physical and physiological characteristics of the schoolchildren studied.

Age (Years)		Weight (kg)		Standing Height (cm)		BMI (kg/m^2^)		WC (cm)		Steps (Number)		Average HR (bpm)		Maximum HR (bpm)	
	*n*	X	SD	X	SD	X	SD	X	SD	X	SD	X	SD	X	SD
Males (*n* = 292)															
6–7 years	19	25.0	3.1	121.6	6.1	16.7	1.5	60.7	4.4	1423.9 *	310.1	152.8	16.6	173.1	15.2
8–9 years	37	33.2	9.4	131.4	6.7	19.0	3.5	68.4 *	11.9	1244.2 *	538.5	149.1	17.1	172.9	15.3
10–11 years	22	41.0	13.0	143.1	8.9	19.8	4.7	73.2	11.9	1054.1 *	423.5	148.8	17.6	171.6	17.0
12–13 years	64	51.7	9.8	155.4 *	8.2	21.4	3.5	74.0 *	9.0	1087.7 *	419.1	124.1	17.8	151.0	19.8
14–15 years	106	60.0 *	11.7	163.3 *	6.4	22.4	4.0	77.5 *	10.5	707.5	400.9	105.1	15.2	128.6	18.2
16–17 years	44	63.1 *	10.2	165.6 *	7.4	23.0	3.5	78.5 *	8.6	605.6	344.5	100.9	14.5	125.1	17.0
Females (*n* = 202)															
6–7 years	20	24.2	7.1	119.4	5.4	17.2	4.5	63.1	12.8	1210.0	363.0	147.2	9.7	171.8	10.1
8–9 years	27	30.3	7.9	130.5	7.2	18.5	3.4	61.8	9.2	1372.0	287.3	142.3	8.8	164.2	15.3
10–11 years	24	42.8	10.1	145.3	6.5	20.1	3.8	72.6	9.8	940.3	369.1	137.1	17.9	162.5	19.1
12–13 years	42	51.4	9.6	152.5	5.7	22.0	3.4	70.4	8.4	853.2	389.7	118.0	16.4	142.1	17.8
14–15 years	57	56.0	11.6	154.5	4.4	23.4	4.6	73.4	10.1	667.7	402.1	104.5	15.7	127.3	14.7
16–17 years	32	58.2	8.5	156.2	5.3	23.8	3.4	75.4	7.8	583.8	266.5	100.2	10.2	123.8	12.5

Legend: X: mean, SD: standard deviation, PI: weight index, WC: waist circumference, HR: heart rate, *: significant difference in relation to women (*p* < 0.05).

**Table 2:** Percentile distribution of the number of steps taken in 15 min school breaks by age range and sex.

Age (Years)	L	M	S	P3	P5	P10	P15	P25	P50	P75	P85	P90	P95	P97
Males														
6–7 years	1.34	1406.4	0.25	656.6	762.9	917.4	1016.8	1157.8	1406.4	1640.9	1762.0	1842.5	1959.6	2034.4
8–9 years	1.08	1288	0.35	407.0	522.5	696.5	812.0	980.2	1288.0	1590.0	1750.2	1858.0	2016.9	2119.6
10–11 years	0.84	1108.8	0.42	305.4	396.5	543.5	646.5	803.4	1108.8	1428.3	1604.8	1726.0	1908.3	2028.1
12–13 years	0.6	977.5	0.48	263.7	333.0	451.8	539.8	681.0	977.5	1314.9	1512	1651.7	1867.5	2013.2
14–15 years	0.35	656.2	0.56	178.6	218.1	289.2	344.9	439.1	656.2	933.3	1109	1239.4	1450.4	1599.1
16–17 years	0.14	515.5	0.62	143.7	170.9	221.4	262.3	334.5	515.5	775.0	955.8	1097.8	1341.7	1523.9
Females														
6–7 years	1.89	1319.5	0.20	683.3	788.2	929.0	1014	1129.1	1319.5	1488.1	1571.8	1626.4	1704.3	1753.3
8–9 years	1.39	1241.4	0.29	462.4	579.9	744.6	848.0	992.2	1241.4	1472.3	1590.4	1668.5	1781.6	1853.6
10–11 years	0.92	1029.0	0.37	337.0	420.2	550.8	640.4	774.4	1029.0	1288.7	1429.8	1526.0	1669.4	1763.1
12–13 years	0.52	802.0	0.46	258.4	310.7	400.1	466.6	573.8	802.0	1066.8	1223.9	1336.2	1511.3	1630.7
14–15 years	0.24	613.3	0.54	191.5	225.9	287.7	336.2	418.9	613.3	869.7	1037.2	1163.8	1372.7	1522.8
16–17 years	0.08	504.0	0.62	146.8	172.6	220.5	259.4	328.4	504.0	762.3	946.5	1093.5	1350.4	1545.7

Legend: P: percentile, L: (skewness, lambda), M: (median, mu), S: (coefficient of variation, sigma).

**Table 3:** BMI and WC values according to step categories in a school recess.

Indicators	Categories of Number Steps						Comparisons			
	Under Average		Average		Above Average		*p*	UA-A	UA-AA	A-AA
	X	SD	X	SD	X	SD				
Males										
*n*	82		124		86					
BMI (kg/m^2^)	21.9	5.0	20.6	4.2	20.1	4.4	0.01	>0.05	<0.05	>0.05
WC (cm)	78	12.2	74.3	10.5	74.1	9.6	0.027	<0.05	<0.05	>0.05
Females										
*n*	55		89		58					
BMI (kg/m^2^)	22.0	3.3	20.9	4.6	19.6	5.3	0.05	>0.05	<0.05	>0.05
WC (cm)	74.5	7.1	71.1	7.8	69.5	9.5	0.004	<0.05	<0.01	<0.05

Legend: BMI: body mass index, X: mean, SD: standard deviation, BP: below average (<p25), *p*: average (p25 to p75), AP: above average (>p75).

## Data Availability

The data is available upon request from the authors.
